# An ontology-based approach for harmonization and cross-cohort query of Alzheimer’s disease data resources

**DOI:** 10.1186/s12911-023-02250-z

**Published:** 2023-08-04

**Authors:** Xubing Hao, Xiaojin Li, Guo-Qiang Zhang, Cui Tao, Paul E. Schulz, The Alzheimer’s Disease Neuroimaging Initiative, Licong Cui

**Affiliations:** 1 McWilliams School of Biomedical Informatics, The University of Texas Health Science Center at Houston, Houston, TX USA; 2 Department of Neurology, McGovern School of Medicine, The University of Texas Health Science Center at Houston, Houston, TX USA; 3 Texas Institute for Restorative Neurotechnologies, The University of Texas Health Science Center at Houston, Houston, TX USA

**Keywords:** Alzheimer’s disease, Ontology, Data element mapping, Data harmonization, Cross-cohort query

## Abstract

**Background:**

In the United States, the National Alzheimer’s Coordinating Center (NACC) and the Alzheimer’s Disease Neuroimaging Initiative (ADNI) are two major data sharing resources for Alzheimer’s Disease (AD) research. NACC and ADNI strive to make their data more FAIR (findable, interoperable, accessible and reusable) for the broader research community. However, there is limited work harmonizing and supporting cross-cohort interoperability of the two resources.

**Method:**

In this paper, we leverage an ontology-based approach to harmonize data elements in the two resources and develop a web-based query system to search patient cohorts across the two resources. We first mapped data elements across NACC and ADNI, and performed value harmonization for the mapped data elements with inconsistent permissible values. Then we built an Alzheimer’s Disease Data Element Ontology (ADEO) to model the mapped data elements in NACC and ADNI. We further developed a prototype cross-cohort query system to search patient cohorts across NACC and ADNI.

**Results:**

After manual review, we found 172 mappings between NACC and ADNI. These 172 mappings were further used to construct common concepts in ADEO. Our data element mapping and harmonization resulted in five files storing common concepts, variables in NACC and ADNI, mappings between variables and common concepts, permissible values of categorical type data elements, and coding inconsistency harmonization, respectively. Our cross-cohort query system consists of three core architectural elements: a web-based interface, an advanced query engine, and a backend MongoDB database.

**Conclusions:**

In this work, ADEO has been specifically designed to facilitate data harmonization and cross-cohort query of NACC and ADNI data resources. Although our prototype cross-cohort query system was developed for exploring NACC and ADNI, its backend and frontend framework has been designed and implemented to be generally applicable to other domains for querying patient cohorts from multiple heterogeneous data sources.

## Background

Alzheimer’s disease (AD) is a neurodegenerative disease affecting over 5.5 million Americans with significant economic and social impacts [1]. It has received a great deal of attention from biomedical research community. In the United States, two major data sharing resources for AD research are the National Alzheimer’s Coordinating Center (NACC) [2] and the Alzheimer’s Disease Neuroimaging Initiative (ADNI) [3]. NACC and ADNI strive to make their data more findable, interoperable, accessible and reusable (FAIR) for the broader research community [4, 5]. They provide valuable resources for discoveries such as AD biomarkers [6], disease progression [7], and cross-cohort model validation [8, 9].

Cross-cohort comparisons allow the research findings obtained from one study to be tested and replicated by another study [10]. Harmonization of heterogeneous data from different resources is essential to enable such cross-cohort comparisons. In addition, harmonizing and integrating data from multiple sources increase the statistical power that an individual dataset would provide. There have been various efforts for cross-cohort data harmonization and integration [11–13] and cross-cohort data exploration [14–17] in different disease domains. For instance, Cui et al. have performed data harmonization on heterogeneous datasets in the the National Sleep Research Resources (NSRR) and developed a cross-cohort search interface called X-search for querying patient cohorts from multiple datasets in NSRR [17].

However, there has been limited work harmonizing AD-related datasets from different resources (such as NACC and ADNI) and supporting cross-cohort data exploration from these AD-related data resources. In a recent preprint [18], Salimi et al. manually harmonized 1,196 variables across 20 AD cohort datasets including NACC and ADNI, and presented a web-based platform called ADataViewer to explore the cohort datasets. However, their data harmonization only mapped a sub-collection of variables (or data elements) from each dataset, and did not harmonize inconsistent codes (or permissible values); in addition, the web-based data exploration was provided in a summarized manner (e.g., pre-computed variable distribution plots), and only supported variable-level queries (e.g., number of patients with variable “Mini-Mental State Examination (MMSE)” captured) rather than value-level queries (e.g., number of patients with MMSE below 12).

In this work, we focused on mapping data elements in NACC and ADNI, and harmonizing inconsistent codes such as different values ranges or different units of measurement for mapped data elements. For data exploration, we developed a prototype cross-cohort, value-level query system for searching patient cohorts across the two resources. In particular, we created an Alzheimer’s Disease Data Element Ontology (ADEO), which not only models harmonized data elements between NACC and ADNI, but also serves as the knowledge source to drive the cross-cohort query system.

### NACC and ADNI

NACC has been collecting data from Alzheimer’s Disease Research Centers (ADRCs) funded by the National Institute on Aging since 2005 [2]. The goal is to translate research advances into improved diagnosis and care for AD patients, and find ways to treat and possibly prevent Alzheimer’s disease and related dementias (ADRD). It includes participants with cognitive status ranging from normal cognition, to mild cognitive impairment (MCI), and demented. In each participant’s annual Uniform Data Set (UDS) visit, standardized clinical data consisting of 16 forms are collected, covering topics including subject demographics, neurological examination findings, and diagnosis. Subsets of UDS subjects have imaging data, and cerebrospinal fluid (CSF) biomarker data, genetic data, and autopsy data.

ADNI began in 2004, and its goal is to detect AD at the earliest possible stage, identify ways to track the disease progression with biomarkers, and support advances in AD intervention, prevention, and treatment [3]. The participants include AD patients, mild cognitive impairment subjects, and elderly controls. The data types that ADNI collects include clinical (demographics, physical examinations, and cognitive assessment data), genetic, MRI image, PET image, and biospecimen (blood, urine, and CSF). ADNI data has been used by AD researchers around the world resulting in over 2,100 publications [19].

### Related work on data element mapping and harmonization

Data element harmonization across cohorts has been an active research area for improving the interoperability across different datasets. For example, Pathak et al. mapped phenotype data elements from five Electronic Medical Records and Genomics (eMERGE) Network sites to the National Cancer Institute (NCI) Cancer Data Standards Registry (caDSR) [20]. Liu et al. mapped data elements in the Dental Information Model to the caDSR common data elements [21]. Tao et al. developed a web-based interactive mapping interface for users to find mappings of variables from the North American Association of Central Cancer Registries (NAACCR) data dictionary to the National Cancer Institute (NCI) Thesaurus (NCIt) [22]. In a preprint [18], Salimi et al. created a variable mapping catalog that harmonized 1,196 unique variables in 20 AD cohort datasets through meticulous manual curation.

### Related work on cross-cohort data exploration

There have been query systems developed for searching patient cohorts across different data sources [14–17]. For example, Weber et al. [14] developed the Shared Health Research Information Network (SHRINE) based on i2b2 [23] to query patient cohorts from multiple data sources. Zhang et al. [15] designed and implemented VISual AGgregator and Explorer (VISAGE) for querying across disparate databases in clinical research. Bache et al. [16] defined and validated an adaptable architecture for identifying patient cohorts from multiple heterogeneous data sources. Cui et al. [17] developed an open access interface for querying patient cohorts across nine datasets in NSRR.

## Methods

**Fig. 1:**
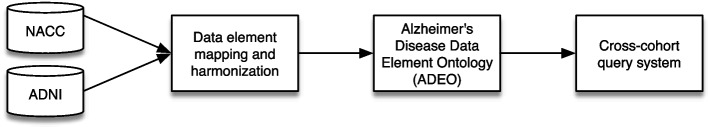
Workflow of our ontology-based approach

Figure 1 shows the overall workflow of our ontology-based approach. After acquiring data from NACC and ADNI, we performed mapping and harmonization of data elements between the two resources. Then we constructed ADEO to formalize the harmonized data elements, which were further leveraged to develop the web-based cross-cohort query system.

### Datasets

We requested study data stored in the format of comma-separated values (CSV) from NACC and ADNI. Each patient may have multiple visits recorded in the study data. NACC stores their study data in a single file, while ADNI separates their study data in different tables. In addition, we downloaded structured data dictionaries (in CSV and PDF) that semantically define the scope and characteristics of data elements (or variables) in the study data. For NACC, the data dictionaries of Uniform Data Set (UDS), Neuropathology (NP) data set, and genetic data are stored in CSV, while the data dictionaries of the imaging and biomarker data sets are stored in PDF. We converted PDF data dictionaries to plain text files using the *pdftotext* utility (part of the *Xpdf* software suite [24]). Then we parsed the plain text files and extracted attributes of data elements and stored them in CSV.

Essential attributes of data elements in NACC’s data dictionary include variable name, form, short descriptor, data type and allowable codes. Essential attributes of data elements in ADNI’s data dictionary include FLDNAME, TEXT, CRFNAME, TYPE and CODE. Variable name in NACC and FLDNAME in ADNI serve as the column name in the study data. Form in NACC and CRFNAME in ADNI are the broader category of each data element. Short descriptor in NACC and TEXT in ADNI store the full name of the data element and are displayed to users in the query interface. Data type in NACC and TYPE in ADNI demonstrate the data type of the data element such as numerical or categorical. Allowable codes in NACC and CODE in ADNI store the permissible values of the categorical type data element or the range of the numerical type data element. Table 1 shows three examples of data elements in NACC’s data dictionary. Table 2 shows three examples of data elements in ADNI’s data dictionary.

**Table 1:** Examples of data elements in NACC

VariableName	Form	VariableType	ShortDescriptor	DataType	AllowableCodes
EDUC	a1	Original UDS question	Years of education	Numeric cross-sectional	0 - 36; 99 = Unknown
NORMCOG	d1	Original UDS question	Normal cognition and behavior	Numeric longitudinal	0 = No; 1 = Yes
NACCVASC	np	NACC Derived Variable	Ischemic, hemorrhagic, or vascular pathology present	Numeric cross-sectional	0 = No; 1 = One or more vascular pathology; 9 = Unknown

**Table 2:** Examples of data elements in ADNI

Phase	FLDNAME	TBLNAME	CRFNAME	TEXT	TYPE	LENGTH	CODE	UNITS
ADNI1	GDAFRAID	GDSCALE	Geriatric Depression Scale	6. Are you afraid that something bad is going to happen to you?	N	1	1=Yes(1); 0=No(0)	
ADNI1	ST127SV	UCSFFRESFR	Longitudinal FreeSurfer	Volume (WM Parcellation) of ThirdVentricle	N	8		mm3
ADNIGO	PTDOBMM	PTDEMOG	Participant Demographics	2a. Participant Month of Birth	N	2	1..12	

### Data element mapping and harmonization

We conducted manual data element mapping to identify overlaps between NACC and ADNI. We first did some pre-processing on the data dictionaries. In NACC’s data dictionaries, the attribute form of the data element is stored using short names (e.g., “a1”, “a2”, “b6”). We converted the short names to their full names provided on NACC’s website for data element mapping. For example, form “a1” has a full name of “Subject Demographics”. In ADNI’s data dictionary, for some imaging-related data elements, words in phrases describing brain regions are concatenated without spaces. We pre-processed such cases and added spaces between the concatenated words. For example, data element “*Cortical Thickness Average of LeftIsthmusCingulate*” in ADNI after pre-processing is “*Cortical Thickness Average of Left Isthmus Cingulate*”. The manual data element mapping were initially performed by XH (expertise in biomedical informatics) and further reviewed by LC and CT (with expertise in biomedical data science and ontology) as well as PES (clinical expert in AD). Disagreements were resolved through discussion.

A unique challenge in exploring data in multiple heterogeneous data sources is to address the coding inconsistency issue, which involves the detection and harmonization of inconsistencies among the disparate permissible values (or value domains) for the same data element  [17]. Such inconsistencies occur frequently for numerical and categorical variables. For example, the permissible values of concept “*Banked postmortem CSF*” is inconsistent between NACC and ADNI. Table 3 shows how we handle the harmonization of the inconsistency for this data element. For the inconsistency of numerical concepts such as “*Years of education*” has a range of “*0 - 36*” in NACC and “*0 - 20*” in ADNI. We always kept the wider range, which is “*0 - 36*” in this case. To ensure accurate results for data exploration, we manually harmonized such heterogeneity.

**Table 3:** Harmonizing coding inconsistencies for data element “*Banked postmortem CSF*”

Source	Value	Name	Value - Harmonized	Name - Harmonized
ADNI	1	Yes	1	Yes
ADNI	2	No	0	No
NACC	0	No	0	No
NACC	1	Yes	1	Yes
NACC	9	Missing/unknown	9	Missing/unknown
NACC	-4	Not available	-4	Not available

We further pre-processed the study data to address other types of inconsistencies before importing them to the database of the cross-cohort query system. For instance, concept “*Segmented right hippocampus volume (cc)*” in NACC uses unit “*cc*” but ADNI uses unit “*mm3*”. Since 1 cc = 1000 mm3, all data points of this data element in ADNI were divided by 1000. Also, for data element “*Average number of packs smoked per day*”, in NACC it has a categorical data type with permissible values “*0 = No reported cigarette use; 1 = 1 cigarette to less than* $\frac{\mathit{1}}{\mathit{2}}$  *pack; 2 =* $\frac{\mathit{1}}{\mathit{2}}$  *pack to less than 1 pack; 3 = 1 pack to 1*$\frac{\mathit{1}}{\mathit{2}}$  *packs; 4 = 1*$\frac{\mathit{1}}{\mathit{2}}$  *packs to 2 packs; 5 = More than two packs; 8 = Not applicable; 9 = Unknown; - 4 = Not available*”, while in ADNI it has a numerical data type with a range of 0 to 10. We grouped the numeric values of this data element in ADNI according to NACC’s categories and stored the categorical results as a new column in the study data.

### ADEO construction

We used the Protege OWL editor (Version 5.5.0) [25] and Owlready2 [26] for building ADEO. Owlready2 is a Python package for manipulating ontologies in the format of Web Ontology Language (OWL). It not only provides the functionality of loading, modifying, and saving ontologies, but also supports reasoning via HermiT [26]. Owlready2 allows a transparent access to OWL ontologies. We used the “types”, which is a Python module to create classes and subclasses dynamically. Subclasses can be created by inheriting an ontology class. Multiple inheritance is also supported.

**Fig. 2:**
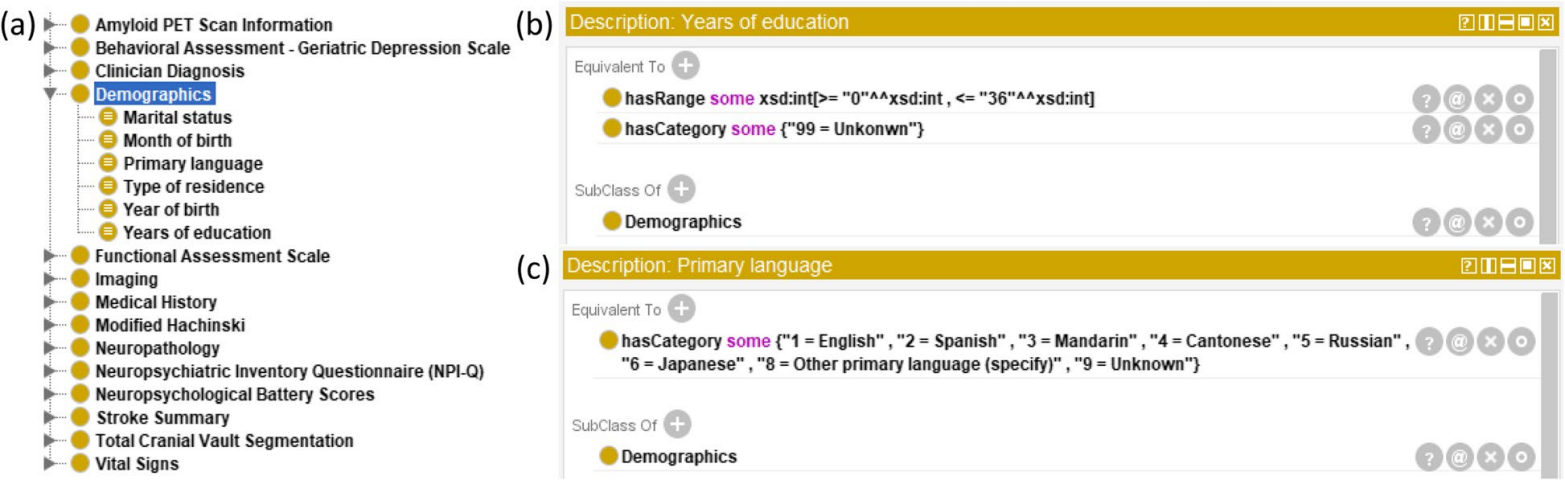
Examples of ADEO classes

We constructed ADEO based on the mapped data elements that we identified from the manual mapping. We defined ADEO classes (or common concepts) to represent the mapped data elements. We created data property classes “hasCategory” and “hasRange” under class “DataProperty” to model permissible values for categorical concepts and specify the range for numerical concepts, respectively. And we used “some” restriction that Owlready2 provided when defining classes. Permissible values and ranges were leveraged to define the classes. We organized the classes into different sub-hierarchies. Figure 2(a) shows the sub-hierarchies of ADEO and the classes under sub-hierarchy “*Demographics*”, including “*Marital status*”, “*Month of birth*”, “*Year of birth*”, “*Primary language*”, “*Type of Residence*”, and “*Years of education*”. Figure 2(b) shows the range defined for class “*Years of education*”. Figure 2(c) shows the categories of permissible values defined for class “*Primary language*”.

In addition, the ADEO classes (or common concepts) will serve as the core query terms in the query system for users to browse or search. Since an ADEO class may correspond to different variable names in NACC and ADNI, there is a need for mapping data elements from NACC and ADNI to the common concepts. Take the common concept “*Large arterial infarcts present*” as an example, its mapped data element in NACC is “*Large arterial infarcts present*” and its mapped data element in ADNI is “*Are one or more large artery cerebral infarcts present?*”.

### Cross-cohort query system development

**Fig. 3:**
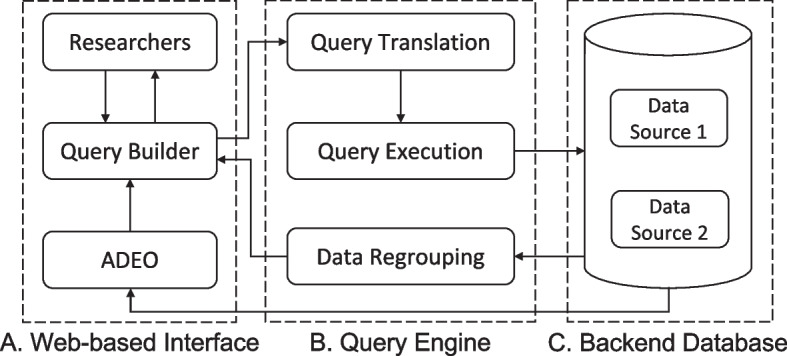
The system architecture of our cross-cohort query system

Figure 3 shows the general architecture design of our cross-cohort query system, consisting of 3 core architectural elements:

1. a web-based interface (see Fig. 3.A), called query builder, which is a powerful and intuitive interface that has been designed and developed to enable researchers to quickly find the right common concepts and perform an exploratory cross-cohort query;
2. an advanced query engine for searching records across different datasets (see Fig. 3.B); such a query engine translates the user queries built from the web-based interface into executable database query languages, and consists of three modules: a query translation module, a query execution module, and data regrouping module; and
3. a backend MongoDB database for storing ADEO and data from different sources (see Fig. 3.C).

We built the query builder interface using React [27], an open-source JavaScript library that is used for building user interfaces specifically for web-based applications. The cross-cohort query engine was implemented using Ruby on Rails [28], which is an agile web development framework.

The query builder consists of four areas, which correspond to four steps to perform cross-cohort queries as follows: 1) dataset selection area, where researchers can select dataset(s) of interest; 2) query term selection area, where users can find and select query terms (i.e., common concepts) and add them to the query construction area; 3) query construction area, where query criteria can be specified for each query term; and 4) query results display area, where the patient counts retrieved from selected dataset(s) satisfying the query criteria are returned to the user.

In this work, the dataset selection area is filled with the names of the two datasets: ADNI and NACC. In the query term selection area, two modes are provided to find query terms of interest: browsing and searching. The browsing mode displays query terms in a hierarchical order, allowing users to explore all accessible query terms level by level. For users with background knowledge, the search mode provides the functionality of directly searching for query terms of interest. Based on the query terms selected by a user, the query builder automatically generates visual query widgets using a dynamic approach, such as generating widgets with checkboxes for specifying permissible values when selecting a categorical term, while creating widgets with sliders for specifying a range of values when selecting a numerical term. The query construction area is designed to be as close to natural language as possible to make the query logic clear and readable to the users. The query results display area is driven by the query criteria specified in the query construction area.

As users add query terms to define queries, the interface creates an array of key-value pairs in JSON objects representing the current state of the user interface and query criteria. Such objects themselves do not contain query language, but instead, contain the query terms as well as additional metadata that describes the query. The query translation module automatically translates the JSON objects into actual MongoDB statements to query the backend database.

The translation relies on the specified query terms and values, as well as the mappings from the data elements in NACC and ADNI to the common concepts representing the query terms. The query statements for disparate data sources are distinct since these data sources have different variable information mapping to a common concept. We have two mapping files that are specifically designed for query term mapping and query value mapping. For example, the query term “Difficulty or need help with: Playing a game of skill” is mapped to the variable “GAMES” in NACC and variable “FAQGAME” in ADNI. The value of “Requires assistance” of this query term is mapped to “2” in NACC and “4” in ADNI.

For each type of query terms, a general template is predefined and used for dynamically generating the actual MongoDB statement for query translation. For instance, the template for querying a numerical query term with a specified range [min, max] is defined as:

- db.records_collection.distinct(<mapped_patient_identifier>,
- {"dataset":<mapped_dataset>,
- <mapped_variable_name>:{"$gte":min, "$lte":max}})

where <mapped_patient_identifier> represents the variable name of the unique patient identifier in the mapped dataset, <mapped_dataset> represents the name of the mapped dataset, and <mapped_variable_name> is the variable name to which the query term is mapped in a dataset. For instance, to query the number of patients with years of education between 5 and 15 years in NACC can be translated to:

- db.records_collection.distinct("NACCID", {"dataset":"NACC", "EDUC":{"$gte":5, "$lte":15}})

The query execution module sends the translated MongoDB statements to the backend database to execute the query. The MongoDB backend returns numeric counts of eligible patients satisfying the query criteria. The data regrouping module summarizes and reorganizes the query results to facilitate the user interface display.

## Results

### Data element mapping and harmonization

The data dictionaries that we downloaded contain 1,195 NACC data elements and 13,918 ADNI data elements. After manual review, we found 172 mappings between NACC and ADNI. Among these mappings, 23 of them required numerical harmonization, 26 of them required a categorical harmonization, and 7 of them required a unit harmonization. These 172 mapping were further used to construct common concepts in ADEO. The core concepts capture information regarding Demographics (e.g., Year of birth, Marital status), Medical History (e.g., Average number of packs smoked per day), vital signs (e.g., Seated Blood Pressure: Systolic), Behavioral Assessment - Geriatric Depression Scale (e.g., Do you feel full of energy?), Modified Hachinski (e.g., Somatic Complaints, Focal Neurologic Signs), Neuropathology (e.g., Banked frozen brain, PRNP codon 129, FTLD-tau subtype - Pick’s (PiD)), Neuropsychological Battery Scores (e.g., Multilingual Naming Test (MINT) - Semantic cues: Number given), Neuropsychiatric Inventory Questionnaire (NPI-Q) (e.g., Anxiety severity, Delusions severity), Functional Assessment Scale (e.g., In the past four weeks, did the subject have any difficulty or need help with: Preparing a balanced meal), Imaging (e.g., Left lingual mean cortical thickness (mm)), Stroke Summary (e.g., Total brain white matter hyperintensity volume (cc)), Amyloid PET Scan Information (e.g., Amyloid Imaging radiotracer used), Total Cranial Vault Segmentation (e.g., Total intracranial volume (cc)), and Clinician Diagnosis (e.g., Normal cognition and behavior). Our data element mapping and harmonization resulted in five files storing common concepts, variables in NACC and ADNI, mappings between variables and common concepts, permissible values of categorical type data elements, and coding inconsistency harmonization, respectively.

### ADEO and cross-cohort query system

**Fig. 4:**
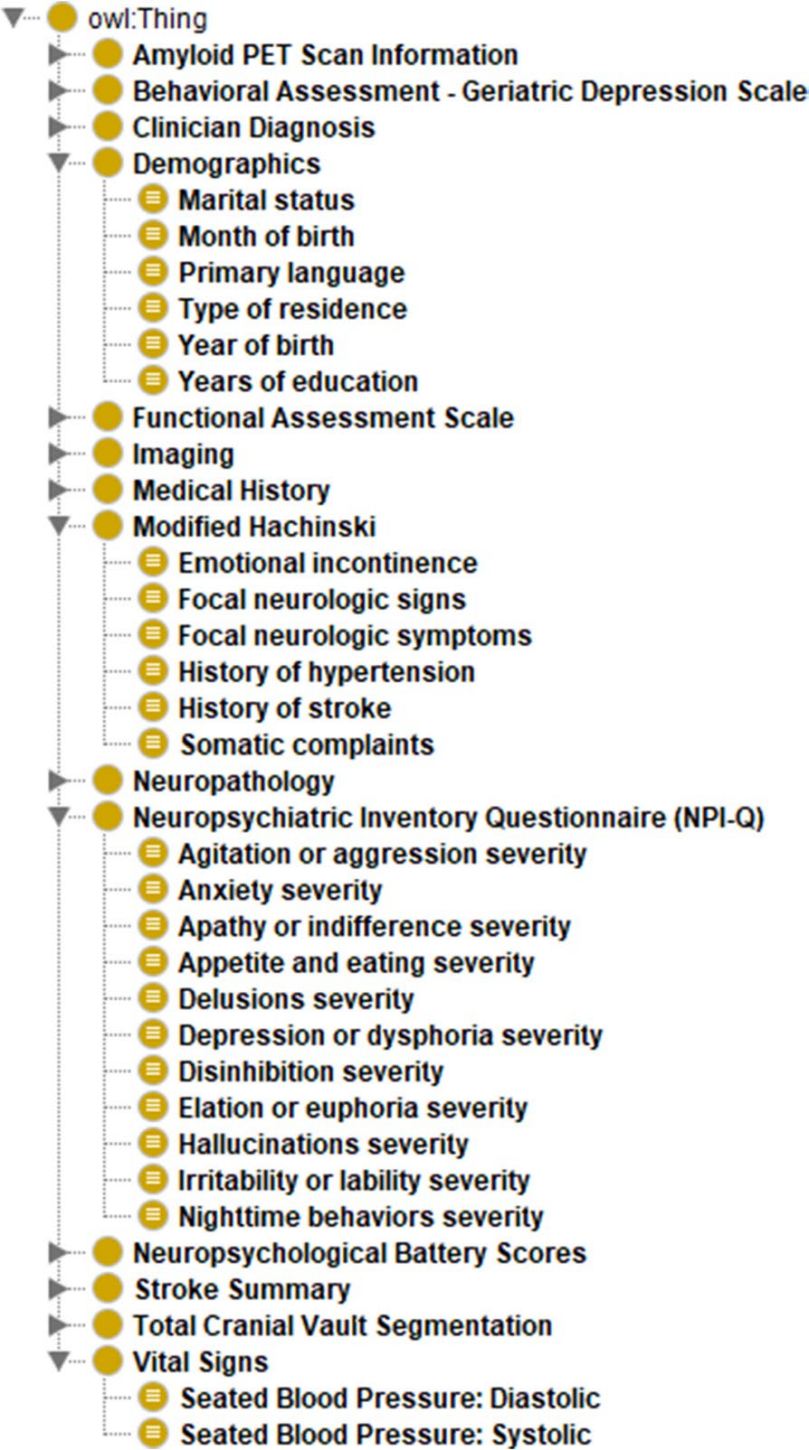
Overall structure of ADEO

The current version of ADEO contains 186 classes. Figure 4 shows the overall structure of ADEO with the following four sub-hierarchies expanded: “*Demographics*”, “*Modified Hachinski*”, “*Neuropsychiatric Inventory Questionnaire (NPI-Q)*”, and “*Vital Signs*”.

**Fig. 5:**
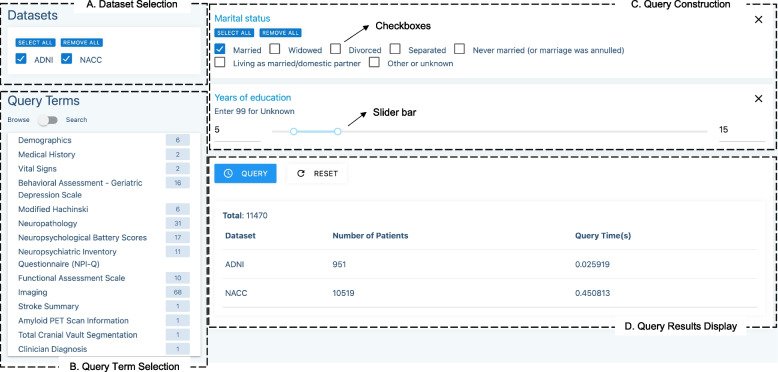
Screenshot of the query builder interface. This example queries the number of married patients with 5 to 15 years of education

The query builder interface with the four areas annotated is shown in Fig. 5. In the dataset selection area (Fig. 5.A), both datasets are chosen. All the core concepts from ADEO are listed in the query term selection area (Fig. 5.B), and researchers can enter text in search mode to obtain the query terms of interest. The query construction area (Fig. 5.C) contains two query widgets for “Marital status” (with checkboxes) and “Years of education” (with a slider bar), with specified query criteria: married, and between 5 and 15 years of education. The query results display area (Fig. 5.D) shows the number of patients satisfying the query criteria in each dataset, as well as the total number of patients satisfying the query criteria across all the selected datasets.

**Fig. 6:**
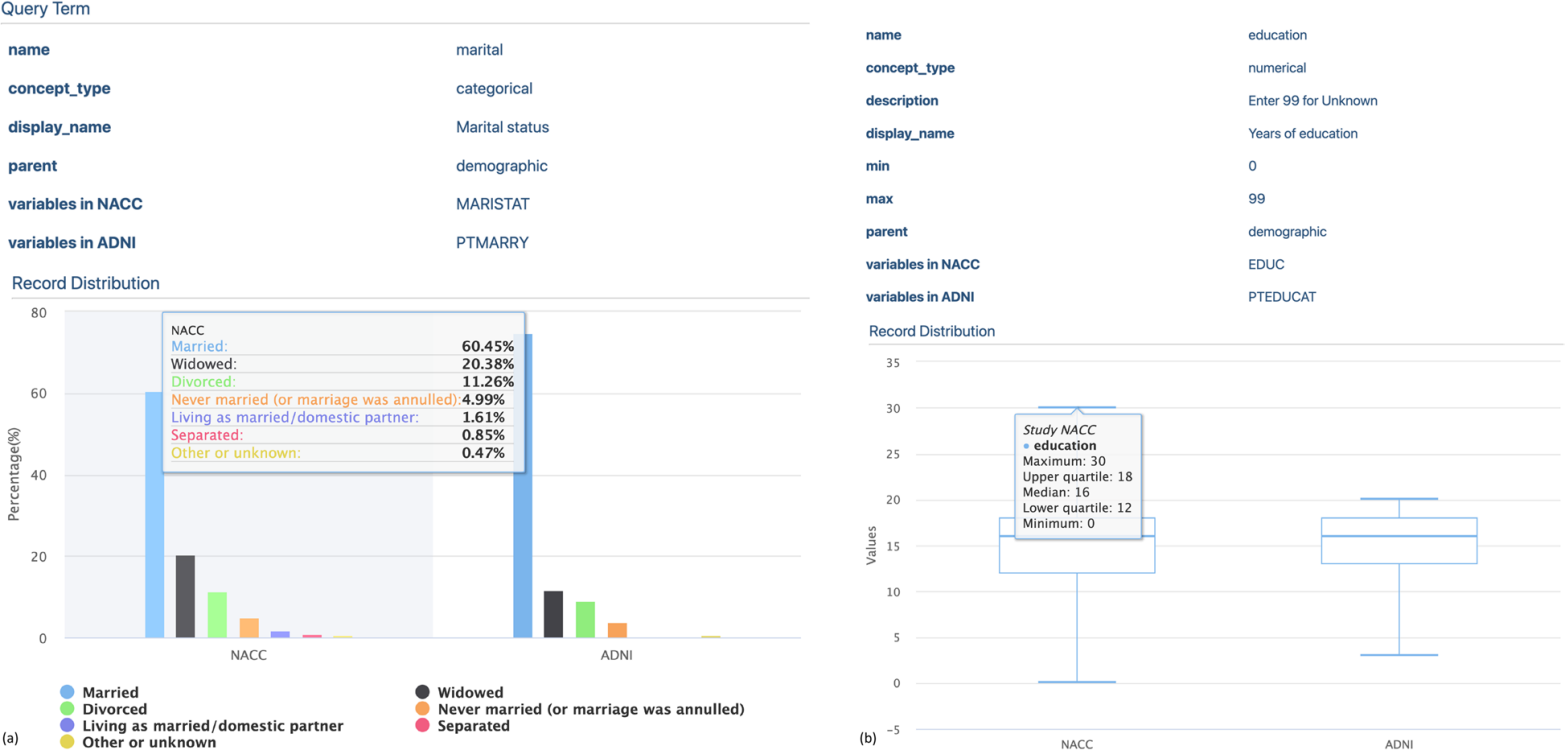
Screenshots of the query term interface: **a** Marital status; and **b** Years of education

To visualize the information for query terms in ADEO, we designed and implemented an interface to display the metadata of each query term. The example of different query term types is shown in Fig. 6, including a categorical query term “Marital status” (Fig. 6(a)) and a numerical query term “Years of education” (Fig. 6(b)). For each type, we generated different interactive visualizations for the distributions of corresponding query term’s values in each dataset, with bar charts for the category type and box plots for the numerical type.

## Discussion

Although our prototype cross-cohort query system was developed for exploring NACC and ADNI, its backend and frontend framework (Figs. 3 and 5) has been designed and implemented to be generally applicable to other domains for exploring patient cohorts from multiple heterogeneous data sources.

### Comparison with related work

In previous studies regarding data element mapping and harmonization, the mapped data elements served different roles in downstream research. For example, Salimi et al. leveraged the harmonized variables to build the interactive ADataViewer to semantically and statistically facilitate the scientific community to explore multiple AD cohort datasets [18]. Tao et al. provided an interface for users to find mappings from data dictionaries to ontologies [22]. Different from these works, we leveraged our harmonized data elements to build a web-based query system for users to search patient cohorts across two widely used AD data resources. Our data element mapping was purely through manual creation to ensure the accuracy of mapping results. We not only mapped variables between data resources but also harmonized their permissible values for our query system purpose.

For the cross-cohort query system, differing from previous work X-search [17] that uses MySQL as the backend database, we choose MongoDB as the backend database in this work considering its good query performance with the large-scale dataset and flexible data models. While X-search stores different data sources in separate MySQL tables, this new system stores all data sources in one collection by leveraging the flexible data model in MongoDB. Such design reduces the complexity of data integration and makes it easier to import data from different sources into the database, and it avoids the need to perform queries across multiple tables. In X-search, the data need to be preprocessed before importing into the MySQL database to handle the coding inconsistency. In this work, we apply a different strategy of creating an additional query value mapping file and mapping inconsistent variable values in real-time in the query translation module; therefore, we do not need to map each inconsistent variable value for all records before importing, thus reducing the tasks and time to build the system.

In addition, although there have been efforts to develop AD-related ontologies such as the Common Alzheimer’s Disease Research Ontology (CADRO) [29] and the Alzheimer’s disease ontology (ADO) [30], these existing AD-related ontologies were not designed (and thus are not sufficient) to be directly usable for supporting harmonizing and querying data elements across different AD data resources. In this work, ADEO has been specifically designed to facilitate such data harmonization and cross-cohort query among different resources.

### Limitations and future work

One limitation of this work is that we did not perform a usability evaluation for the prototype cross-cohort query system. We plan to invite AD researchers to evaluate our prototype query system and provide feedback for us to enhance the system’s functionality and usability. Another limitation of our work is that there may exist missed mappings between NACC and ADNI, even though manual curation was performed. Comparing to Salimi et al.’s work that identified 170 mapped data elements between ADNI and NACC [18], our work identified 172 mapped data elements. Among these mappings, there are 72 identified by both works, 98 identified by Salimi et al.’s work but not ours, and 100 identified by our work but not Salimi et al.’s. We will further incorporate and harmonize those mappings identified by Salimi et al.’s work but not ours. Automated mapping techniques along with manual validation may also help identify further mappings between the two data resources. Additional future work includes enriching ADEO with synonyms leveraging other AD-related ontologies and the Unified Medical Language System.

## Conclusions

In this paper, we presented an ontology-based approach to map and harmonize data elements in NACC and ADNI, two widely used data resources for AD research. We also developed a prototype cross-cohort query system to search patient cohort counts across the two data resources. Our prototype query system is generally applicable to other domains for supporting cross-cohort queries.

## Data Availability

The results for mappings between NACC and ADNI as well as the ADEO ontology are available at https://github.com/XubingHao/BMC2022_AD-Query.

## Abbreviations

NACC: National Alzheimer’s Coordinating Center
ADNI: Alzheimer’s Disease Neuroimaging Initiative
AD: Alzheimer’s Disease
NSRR: National Sleep Research Resources
MMSE: Mini-Mental State Examination
UDS: Uniform Data Set
CSF: cerebrospinal fluid
NCI: National Cancer Institute
caDSR: Data Standards Registry
eMERGE: Electronic Medical Records and Genomics
NP: Neuropathology
OWL: OntologyWebLanguage
CADRO: Common Alzheimer’s Disease Research Ontology
ADO: Alzheimer’s disease ontology
